# Comparative study of femoral neck shortening following two types of internal fixation in young and middle-aged patients with displaced femoral neck fractures

**DOI:** 10.3389/fsurg.2026.1781748

**Published:** 2026-05-11

**Authors:** Yun He, Yiliyaer Abudusimu, Guosheng Wang, Bin Xu, Tayierjiang Yasheng

**Affiliations:** Department of Trauma Orthopedics II, Sixth Affiliated Hospital of Xinjiang Medical University, Urumqi, Xinjiang Uygur Autonomous Region, China

**Keywords:** cannulated screw, femoral neck dynamic cross screw system, femoral neck fracture, femoral neck shortening, FNS

## Abstract

**Objective:**

To compare the outcomes of femoral neck shortening between the Femoral Neck System (FNS) and FNS combined with Cannulated Compression Screws (CCS) in the treatment of displaced femoral neck fractures in young and middle-aged patients.

**Methods:**

A retrospective analysis was conducted on 163 young and middle-aged patients with displaced femoral neck fractures who underwent either FNS or FNS combined with CCS internal fixation surgery in the Department of Trauma Orthopedics at the Sixth Affiliated Hospital of Xinjiang Medical University between September 2019 and January 2023. According to the internal fixation method, patients were divided into the FNS group (*n* = 94) and the combined group (*n* = 69). There were 76 males and 87 females, aged 39 to 61 years, with a mean age of 52.5 years. The causes of injury included 94 cases of road traffic injuries, 31 cases of high falls, and 38 cases of falls. According to the Garden classification, there were 44 cases of type III and 119 cases of type IV. Differences in perioperative indicators, femoral neck shortening, hip joint function, and complications were compared between the two groups.

**Results:**

There were no statistically significant differences between the two groups in terms of age, gender, injury mechanism, injury location, Garden classification, time from fracture to surgery, or the presence of cardiovascular diseases, diabetes, smoking history, or alcohol history (*P* > 0.05). The operative time and intraoperative blood loss in the combined group were significantly greater than those in the FNS group [142 (112, 152) min vs. 81 (76, 92) min; 86 (77, 92) mL vs. 56 (51, 66) mL], with statistical significance (*P* < 0.05). The degree of femoral neck shortening in the combined group was significantly lower than that in the FNS group at 3 months (1.58 ± 0.32 mm vs. 3.04 ± 0.68 mm), 9 months (2.65 ± 0.52 mm vs. 3.98 ± 0.30 mm), and 15 months (2.88 ± 0.79 mm vs. 4.62 ± 1.09 mm) postoperatively, with statistical significance (*P* < 0.05). There were no statistically significant differences between the two groups in reduction quality, postoperative hospital stay, hip Harris score, or complication rates (*P* > 0.05).

**Conclusion:**

Although FNS combined with CCS can reduce the degree of femoral neck shortening in patients with femoral neck fractures, it requires higher surgical expertise, longer operative time, and greater intraoperative blood loss. Surgeons should select the appropriate surgical approach based on the patient's specific circumstances.

## 1 Introduction

Femoralneck fractures represent a common type of orthopedic injury, with global hip fracture cases projected to reach 2.6 million by 2025, approximately 50% of which are femoral neck fractures (1). The incidence of femoral neck fractures in young and middle-aged adults is relatively low, accounting for only 2% to 3% of all femoral neck fractures (2). For patients under 65 years of age, internal fixation is the preferred treatment method (3). Among the various fixation techniques, the use of three parallel cannulated screws is the most common approach (4). However, studies have reported a relatively high failure rate associated with cannulated screws, including complications such as screw loosening, femoral head necrosis, and poor hip joint recovery, highlighting certain limitations of this method (5). The Femoral Neck System (FNS) is a newer fixation method for femoral neck fractures, offering stable anti-rotational and anti-shear forces, which facilitate the recovery of hip joint function (6, 7). Nevertheless, FNS provides only single-plane stability for unstable femoral neck fractures, which may be insufficient to deliver complete and effective mechanical support. Consequently, some patients may experience complications such as hip varus, screw cut-out, loss of fracture reduction, and femoral neck shortening after surgery (8, 9). Among these, femoral neck shortening is the most common complication following internal fixation of femoral neck fractures (10, 11).

While numerous studies have explored the application of FNS in femoral neck fractures, research on femoral neck shortening following FNS combined with supportive cannulated screw fixation remains limited. Therefore, to compare femoral neck shortening outcomes in young patients with displaced femoral neck fractures treated with FNS alone vs. FNS combined with cannulated screws, this study retrospectively analyzed data from 163 young patients with Garden type III and IV femoral neck fractures admitted to our department between September 2019 and June 2023. The findings are reported as follows.

## 2 Subjects and methods

### 2.1 Eneral information

#### 2.1.1 Inclusion criteria

(1) Age between 18 and 65 years; (2) Unilateral femoral neck fracture; (3) Fresh fracture, with the time from injury to surgery ≤ 2 weeks; (4) Treatment with FNS or FNS combined with cannulated screws; (5) Follow-up time ≥ 15 months; (6) Garden type III or IV fracture.

#### 2.1.2 Exclusion criteria

(1) Multiple fractures; (2) Pathological fractures; (3) Congenital hip dysplasia; (4) Incomplete follow-up information.

A total of 163 patients were included in this study. According to the internal fixation method, they were divided into two groups: the Femoral Neck System group (FNS group, *n* = 94) and the FNS combined with cannulated screws group (combined group, *n* = 69). FNS group comprises patients with adequate posteromedial cortical support after reduction, while the FNS + CCS group includes those with residual posteromedial defects or potential rotational instability.This study was approved by the Medical Ethics Committee of the Sixth Affiliated Hospital of Xinjiang Medical University (Ethics Approval No. LFYLLSC20241223-01), and informed consent was obtained from all patients and their families.

### 2.2 Surgical methods

Patients were administered general anesthesia or spinal anesthesia and placed in a supine position on a traction operating table. After routine disinfection, closed reduction of the fracture was performed under the guidance of a C-arm x-ray machine combined with the traction table (1).

FNS Group: A 5 cm longitudinal incision was made 4–6 cm below the greater trochanter, parallel to the axis of the femoral shaft. The skin, subcutaneous tissue, and iliotibial band were sequentially incised to expose the bone surface. Under the guidance of the FNS guide, a positioning guide pin was inserted (neck-shaft angle of 130°, with the pin tip positioned 5 mm from the subchondral bone of the femoral head). Based on the positioning guide pin, the required depth for the dynamic rod implantation was measured. The guide pin hole was enlarged along the positioning guide pin, and an appropriate FNS dynamic rod and plate (Depuy Synthes, Switzerland) were implanted. Once satisfactory fixation of the dynamic rod and plate was achieved, the positioning guide pin was removed (2).

Combined Group: The FNS implantation procedure was the same as in the FNS group. Subsequently, one appropriately sized 7.3 mm titanium semi-threaded cannulated screw (Depuy Synthes, Switzerland) was implanted in the posteromedial aspect of the femoral neck, parallel to the FNS dynamic rod. This was done to address the mechanical instability of the femoral neck caused by posteromedial cortical defects (2).

### 2.3 Perioperative management and follow-up

Pain was assessed using the Visual Analog Scale (VAS) both preoperatively and postoperatively, with oral or intravenous analgesics administered based on pain intensity. Prophylactic cefazolin was administered within 24 h before and after surgery. Oral intake was initiated upon awakening, with a gradual transition to a regular diet in the absence of gastrointestinal symptoms such as nausea and vomiting. Low-molecular-weight heparin was administered prophylactically for anticoagulation upon admission and discontinued 12 h before surgery, with anticoagulation resumed postoperatively. Quadriceps contraction exercises and ankle pump exercises were performed to prevent deep vein thrombosis. Full weight-bearing was permitted when clinical signs and imaging examinations confirmed fracture healing. Outpatient follow-up was conducted at 1, 3, 9, 12, and 15 months postoperatively.

### 2.4 Outcome measures

#### 2.4.1 Primary outcome

Measures included the degree and grade of femoral neck shortening at 3, 9, and 15 months postoperatively. The grading was as follows: mild (<2 mm), moderate (2–<5 mm), severe (5–10 mm), and extreme (>10 mm).

#### 2.4.2 Secondary outcome

Measures included surgery-related indicators such as operative time, intraoperative blood loss, fluoroscopy frequency, postoperative hospital stay, reduction quality, hip function indicators, and complications.

Calculation Method for Femoral Neck Shortening:

#### 2.4.3 FNS group

**Figure 1:**
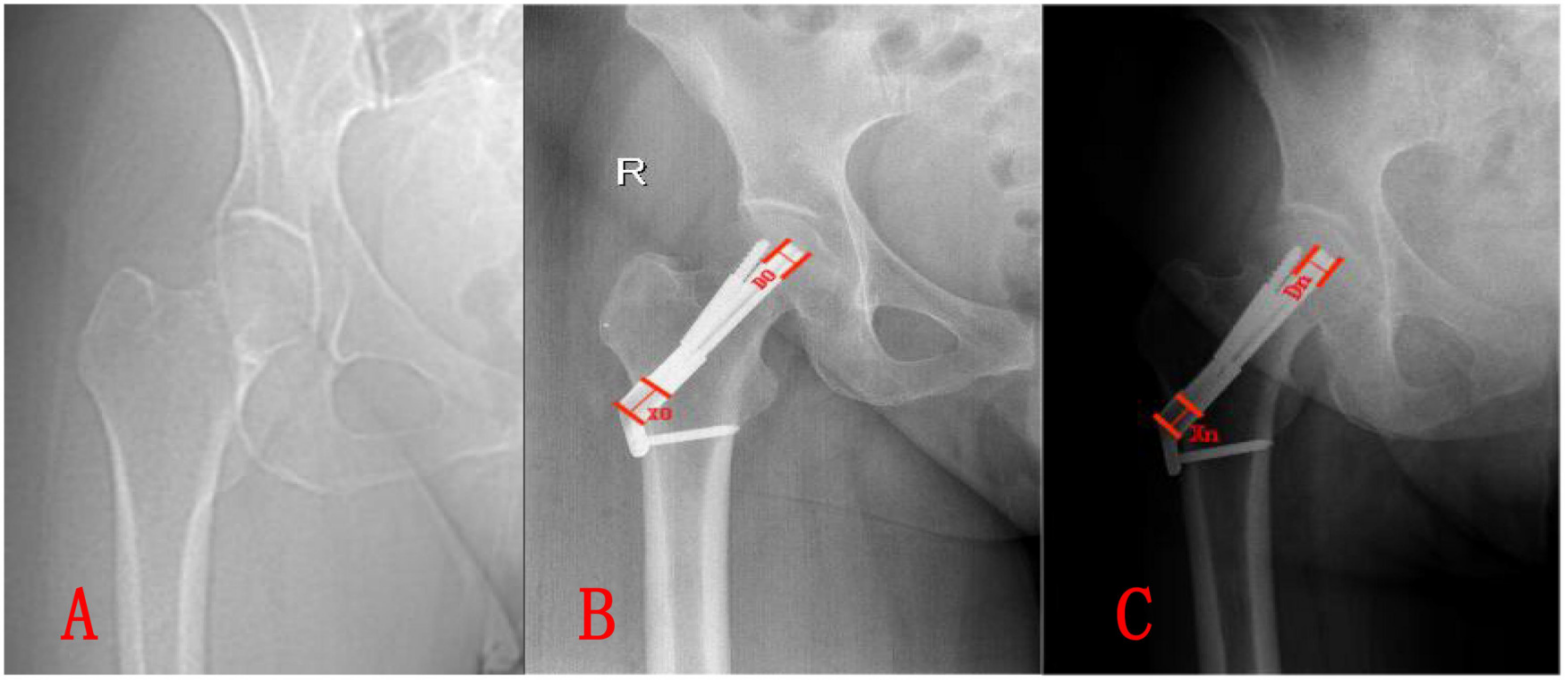
A 43-year-old female patient with a garden type III right femoral neck fracture due to a traffic injury. Open reduction and internal fixation with FNS were performed on the third day after the fracture, following comprehensive examinations. **A**. Admission x-ray showing a complete fracture, classified as Garden type III femoral neck fracture. **B**. Immediate postoperative anteroposterior x-ray after FNS fixation, demonstrating satisfactory fracture reduction and centrally positioned FNS internal fixation. **C**. Follow-up x-ray at 15 months postoperatively, showing blurred fracture lines, acceptable fracture healing, and normal hip function. X₀: distance from the head screw tip to the lateral plate, D₀: bolt diameter, Xₙ, Dₙ: distance from the head screw tip to the lateral plate and bolt diameter measured on the x-ray at 15 months postoperatively, Xᵣ: degree of femoral neck shortening.

Following previously reported measurement methods, two observers measured the distance from the tip of the head screw to the lateral plate on postoperative x-rays (X₀) and the bolt diameter (D₀). On follow-up x-rays, the distance from the head screw tip to the lateral plate (Xₙ) and the bolt diameter (Dₙ) were measured at each follow-up time point (as shown in Figure 1). The actual diameter of the bolt was denoted as Dᵣ. The sliding distance of the bolt tip (Xᵣ) represented the actual femoral neck shortening, calculated using the formula: Xᵣ = (X₀ – Xₙ) × (Dᵣ/D₀) (12, 13).

#### 2.4.4 Combined group

**Figure 2:**
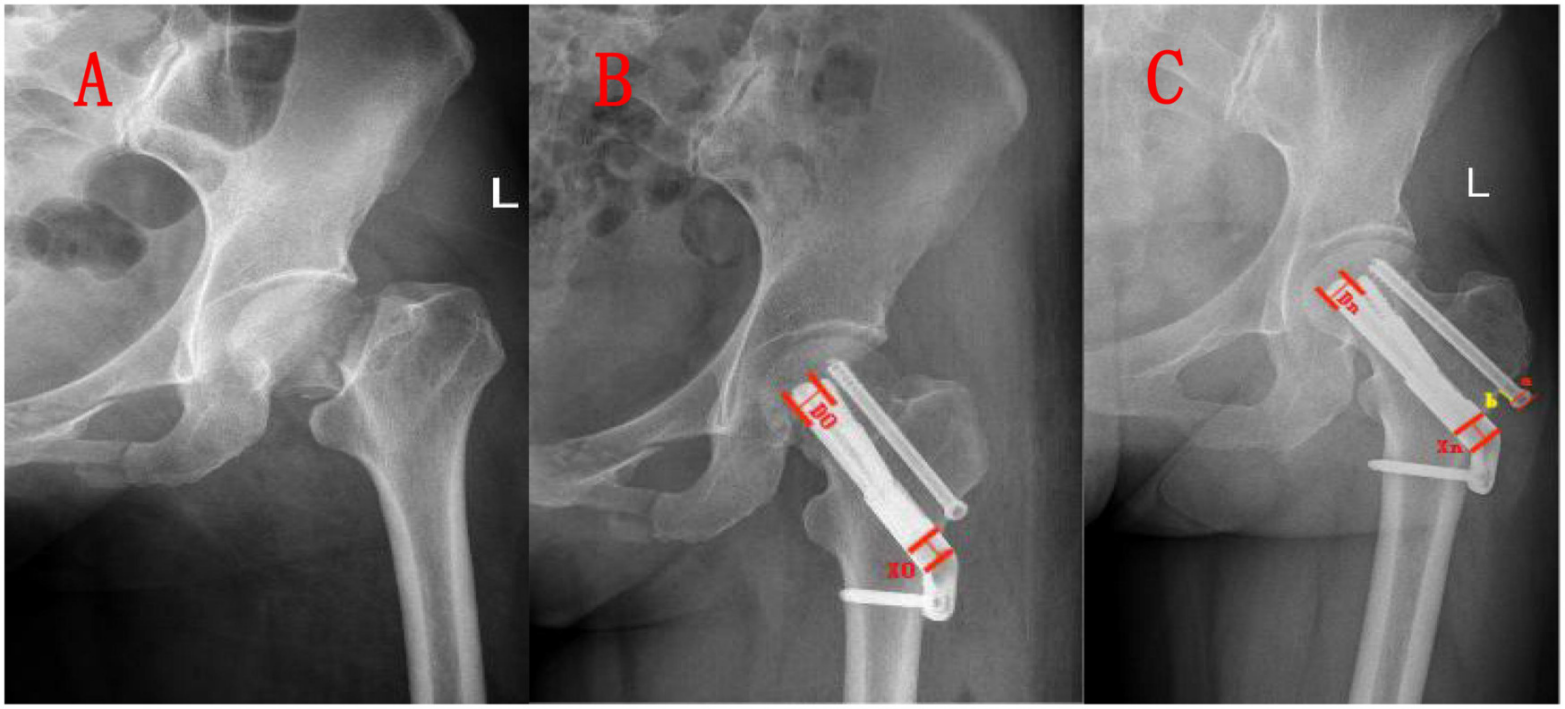
A 55-year-old male patient with a garden type IV left femoral neck fracture caused by a traffic injury. The fracture was complete with displacement of the femoral head. After comprehensive examinations, open reduction and internal fixation with FNS combined with a cannulated screw were performed on the fifth day after the fracture. **A**. Admission x-ray showing a complete fracture with coxa vara and inferior displacement of the femoral head. **B**. Immediate postoperative x-ray after fixation with FNS combined with a cannulated screw, demonstrating acceptable fracture reduction, clear fracture margins, and centrally positioned internal fixation. **C**. Follow-up x-ray at 15 months postoperatively, showing nearly disappeared fracture lines, excellent fracture healing, and normal hip function in the patient. a: screw cap thickness; b: exposed screw length; c: actual thickness of the screw cap.

The measurement for the FNS group was performed as described above. For the cannulated compression screw (CCS), the measurement was based on the method reported by Xia Xi (14) and Li Weilong et al. (15) using the exposed screw length measurement technique. As shown in Figure 2, the vertical distance between the two red lines represents the projected thickness of the screw cap on imaging (a), the yellow line indicates the length of the exposed screw on imaging (b), and the actual thickness of the hollow screw cap is defined as (c). According to the formula femoral neck shortening length = b × c/a, the longer exposed length between the two types of internal fixation was selected as the measurement reference at each timepoint.

### 2.5 Statistical analysis

Statistical analysis was performed using SPSS Statistics 26.0 software. Continuous data such as age, BMI, time from fracture to surgery, and femoral neck shortening at 3, 9, and 15 months postoperatively were tested for normality. If normally distributed, data were expressed as mean ± standard deviation (X ± S) and compared between groups using independent samples t-tests. Continuous data that were not normally distributed, such as operative time, blood loss, postoperative hospital stay, and hip joint scores, were expressed as median (Q1, Q3) and compared using the Mann–Whitney U test. Categorical data, including gender, injury mechanism, injury side, Garden classification, presence of cardiovascular disease, diabetes, smoking history, alcohol history, reduction quality, need for blood transfusion, complications, and femoral neck shortening grade, were reported as counts and percentages. Since the total sample size exceeded 40, chi-square tests were used to compare differences between groups for categorical variables. For repeated measures data, repeated measures ANOVA was applied to compare differences between groups across time points, with *post-hoc* multiple comparisons used to assess differences at specific time points. A *P*-value <0.05 was considered statistically significant.

## 3 Results

### 3.1 General information

There were no statistically significant differences in age, gender, injury mechanism, injury location, Garden classification, time from fracture to surgery, or the presence of cardiovascular disease, diabetes, smoking history, or alcohol history between the two groups (*P* > 0.05), as shown in Table 1.

**Table 1:** Comparison of general data between the two groups of femoral neck fracture patients (n, X ± S).

Item	FNS Group (*n* = 94)	Combined Group (*n* = 69)	t/*χ*2	*P*
Age (years)	52.80 ± 8.95	52.20 ± 8.18	t = 0.418	0.677
Gender (male/female)	44/50	32/37	χ2 = 0.051	0.822
BMI (kg/m2)	23.70 ± 4.05	23.55 ± 5.08	t = 0.210	0.834
Injury mechanism (traffic/fall/tumble)	56/18/20	38/13/18	χ2 = 0.244	0.885
Injury side (left/right)	50/44	42/27	χ2 = 1.156	0.282
Garden classification (III/IV)	26/68	18/51	χ2 = 0.072	0.789
Time from fracture to surgery (days)	4.05 ± 0.81	4.01 ± 0.78	t = 0.032	0.975
Cardiovascular disease (yes/no)	18/76	16/53	χ2 = 0.392	0.531
Diabetes (yes/no)	14/80	10/59	χ2 = 0.005	0.945
Smoking history (yes/no)	21/73	9/60	χ2 = 1.847	0.174
Alcohol history (yes/no)	18/76	9/60	χ2 = 0.881	0.348

### 3.2 Comparison of surgical-related indicators between the two groups

The combined group had significantly longer operative time and greater intraoperative blood loss than the FNS group (*P* < 0.05). There were no statistically significant differences in reduction quality, postoperative hospital stay, blood transfusion rate, hip joint score, thrombosis, pressure sores, or incision-related adverse events between the two groups (*P* > 0.05), as shown in Table 2.

**Table 2:** Comparison of surgical, HHS, and complication-related indicators between the two groups [M(Q1,Q3)].

Item	FNS Group (*n* = 94)	Combined Group (*n* = 69)	Z/χ2	*P*
Operative time (minutes)	81 (76, 92)	142 (112, 152)	Z=−9.024	<0.001
Intraoperative blood loss (mL)	56 (51, 66)	86 (77, 92)	Z=−9.315	<0.001
Reduction quality (anatomical/functional/fair)	58/22/14	40/12/17	χ2 = 0.532	0.766
Postoperative hospital stay (days)	6 (6, 7)	7 (6, 8)	Z=−1.624	0.104

### 3.3 Comparison of the two groups regarding the primary outcome indicators

The combined group showed significantly lower femoral neck shortening compared to the FNS group at 3 months (1.58 ± 0.32 mm vs. 3.04 ± 0.68 mm), 9 months (2.65 ± 0.52 mm vs. 3.98 ± 0.30 mm), and 15 months (2.88 ± 0.79 mm vs. 4.62 ± 1.09 mm) postoperatively (*P* < 0.05). There was no statistically significant difference in the distribution of mild, moderate, severe, or extreme femoral neck shortening between the two groups (*P* > 0.05), as shown in Table 3.

**Table 3:** Comparison of primary outcome indicators between the two groups (n, X ± S).

Item	FNS Group (*n* = 94)	Combined Group (*n* = 69)	t/χ2	*P*
Femoral neck shortening grade (n)			χ2 = 0.287	0.963
<2 mm (mild)	44	32		
2–<5 mm (moderate)	24	16		
5–10 mm (severe)	16	10		
>10 mm (extreme)	10	11		
Postoperative femoral neck shortening (mm)				
3 months	3.04 ± 0.68	1.58 ± 0.32	t = 13.945	<0.001
9 months	3.98 ± 0.30	2.65 ± 0.52	t = 16.812	<0.001
15 months	4.62 ± 1.09	2.88 ± 0.79	t = 9.483	<0.001

### 3.4 Comparison of complication rates between the two groups

There were no statistically significant differences in the incidence of deep vein thrombosis, poor incision healing, pressure sores, or total complications between the two groups (*P* > 0.05), as shown in Table 4.

**Table 4:** Comparison of complication rates between the two groups (n).

Group	Deep vein thrombosis	Poor incision healing	Pressure sore	Total complications
FNS Group (*n* = 94)	4	2	8	14
Combined Group (*n* = 69)	5	1	5	11
χ2	0.284	0.000	0.012	0.155
P	0.594	1.000	0.913	0.694

### 3.5 Comparison of postoperative hip joint harris scores and VAS between the two patient groups

At 1 month and 1 year postoperatively, no statistically significant differences were observed in Harris Hip Scores or VAS pain scores between the two groups (*P* > 0.05). Specifically, the 1-month Harris scores were 44.85 ± 3.58 in the FNS group and 45.02 ± 3.86 in the combined group (t = 0.290, *P* = 0.772), while the 1-year scores were 81.08 ± 6.25 and 82.14 ± 7.58, respectively (t = 0.977, *P* = 0.330). Similarly, the 1-month VAS scores were 3.31 ± 1.07 and 3.22 ± 1.01 (t = 0.536, *P* = 0.592), and the 1-year VAS scores were 1.47 ± 0.62 and 1.36 ± 0.58 (t = 1.144, *P* = 0.254),as shown in Table 5.

**Table 5:** Comparison of hip joint harris scores and VAS between the two groups (X ± S, points).

Group	Follow up time	FNS Group (*n* = 94)	Combined Group (*n* = 69)	t	*P*
HHS	1 Month	44.85 ± 3.58	45.02 ± 3.86	0.290	0.772
	1 Year	81.08 ± 6.25	82.14 ± 7.58	0.977	0.330
VAS	1 Month	3.31 ± 1.07	3.22 ± 1.01	0.536	0.592
	1 Year	1.47 ± 0.62	1.36 ± 0.58	1.144	0.254

## 4 Discussion

Femoral neck fractures account for 3.6% to 6.0% of all fractures, with the vast majority requiring surgery. For patients under 70 years of age with femoral neck fractures, internal fixation is the treatment of choice, and cannulated compression screws (CCS) have long been regarded as the classic internal fixation method for femoral neck fractures (16–18). While cannulated screw fixation offers advantages such as minimal trauma, rapid recovery, short operative time, low cost, and ease of dissemination (19), it still faces technical challenges such as femoral neck shortening (20). The Femoral Neck System (FNS) is a new-generation internal fixation system developed on the basis of traditional CCS and the dynamic hip screw. It provides good anti-rotational and angular stability while allowing minimally invasive insertion and permitting sliding compression at the fracture ends (21). Although the sliding compression mechanism promotes fracture healing and has achieved favorable clinical healing outcomes, excessive sliding can lead to severe femoral neck shortening (22). In routine clinical practice, our research team observed that despite the strong stability offered by FNS, the incidence of femoral neck shortening postoperatively is not uncommon. This finding is largely consistent with the results reported by Zheng et al. (12). Therefore, our team added one semi-threaded cannulated screw to the standalone FNS fixation to explore whether it could reduce the burden of femoral neck shortening, aiming to provide reliable theoretical support for orthopedic surgeons in choosing an internal fixation method.

The results of this study show that the degree of femoral neck shortening at 3, 9, and 15 months postoperatively was significantly better in the combined group than in the FNS group. This may be because adding a cannulated screw to standalone FNS fixation can reduce the screw back-out rate of the FNS, thereby better addressing the occurrence of femoral neck shortening after femoral neck fracture surgery. Vazquez et al. (23) followed up 63 patients over 75 years of age with non-displaced femoral neck fractures and found that the femoral neck shortening length in the FNS group (9.3 ± 6.0 mm) was greater than that in the CCS group (8.4 ± 7.0 mm) at 3 months postoperatively, but the difference was not statistically significant. Xu et al. (24) followed up 105 patients with femoral neck fractures and found no statistically significant difference in the degree of femoral neck shortening between the FNS group and the CCS group. This aligns with the research background of our experimental group, indicating that FNS does not have a better preventive effect on femoral neck shortening compared to CCS. The combination of FNS and cannulated screws is not used for the first time. A recent biomechanical report (25) indicated that FNS combined with cannulated screws for the treatment of displaced femoral neck fractures in young and middle-aged adults provides better biomechanical stability than standalone FNS fixation. Researchers such as Tusongjiang and Ge Shuanglei et al. (10, 11) reported that FNS combined with cannulated screws yields good clinical efficacy in treating displaced femoral neck fractures in young and middle-aged adults. This study shows that the combined group had longer operative times and greater intraoperative blood loss compared to the FNS group. This is consistent with actual clinical practice: adding an extra cannulated screw and placing it near the center of the femoral head not only requires proficient surgical skills from the operator but also increases operative time, fluoroscopy frequency, and trauma.

We believe this is primarily related to the following biomechanical principles: While simple FNS fixation provides angular stability and sliding compression, its single-plane anti-rotation capability is limited in unstable femoral neck fractures with posterior medial cortical defects, making the fracture ends prone to excessive sliding under axial loads and subsequent shortening. An additional posterior medial parallel hollow screw effectively addresses this mechanical deficiency—the screw not only serves as a “medial support screw” to restore the load-bearing function of the posterior medial femoral neck column and distribute the compressive stress transmitted through the femoral trochanter, but also significantly enhances the overall configuration's anti-rotation and anti-shear resistance. This combined fixation limits excessive retraction of the FNS dynamic rod, confining the sliding distance within a physiological range conducive to fracture healing, thereby reducing shortening without compromising compression efficacy. Recent finite element studies have also confirmed that FNS combined with a medial support screw significantly reduces displacement and varus angle at the fracture site.

Limitations of this study:①The sample size of the combined group is relatively small. This is because this internal fixation technique has only recently been adopted, and the number of follow-up cases is limited, which may affect the final results;②The study focused on femoral neck shortening-related indicators in patients but did not consider conditions such as nonunion or femoral head necrosis;③The follow-up period is relatively short, which may affect the results regarding femoral neck shortening and other complications. The follow-up duration should be extended in future studies (4). As a retrospective study, recall bias may exist. Future research should involve larger sample sizes and multicenter studies.

In summary, although FNS combined with cannulated screws can reduce the degree of femoral neck shortening in patients after femoral neck fracture surgery, it requires higher technical expertise from the surgeon, longer operative time, and greater intraoperative blood loss. In clinical practice, surgeons should choose the surgical approach based on the patient's specific circumstances.

## Data Availability

The raw data supporting the conclusions of this article will be made available by the authors, without undue reservation.
